# Gonorrhea and Chlamydia Testing and Case Rates Among Women Veterans in the Veterans Health Administration

**DOI:** 10.1007/s11606-022-07578-2

**Published:** 2022-08-30

**Authors:** Shimrit Keddem, Marissa Maier, Carolyn Gardella, Joleen Borgerding, Elliott Lowy, Maggie Chartier, Sally Haskell, Ronald G. Hauser, Lauren A. Beste

**Affiliations:** 1 Corporal Michael J. Crescenz Veterans Affairs (VA) Medical Center, VA Center for Health Equity Research and Promotion (CHERP), Philadelphia, PA USA; 2 Perelman School of Medicine, University of Pennsylvania, Philadelphia, PA USA; 3 Division of Infectious Diseases, Department of Medicine, Oregon Health & Science University, Portland, OR USA; 4 VA Portland Health Care System, Veterans Health Administration (VHA), Portland, OR USA; 5 VA Puget Sound Health Care System, Veterans Health Administration (VHA), Seattle, WA USA; 6 Department of Obstetrics and Gynecology, University of Washington School of Medicine, Seattle, WA USA; 7 Department of Medicine, University of Washington School of Medicine, Seattle, WA USA; 8 Office of Specialty Care Services, Veterans Health Administration, Washington, DC USA; 9 Pathology and Laboratory Medicine Department, Veterans Affairs Connecticut Health Care, West Haven, USA; 10 Yale University School of Medicine, New Haven, CT USA

**Keywords:** Veterans, sexually transmitted infections (STI), screening, women’s health, gonorrhea, chlamydia

## Abstract

**Background:**

United States (US) rates of sexually transmitted infection (STI) in women, especially gonorrhea and chlamydia, have increased over the past decade. Women Veterans may be at increased risk for STIs due to high rates of sexual trauma. Despite the availability of effective diagnostic tests and evidence-based guidelines for annual screening among sexually active women under age 25, screening rates for gonorrhea and chlamydia remain low in the US and among Veterans.

**Objective:**

To examine patient characteristics and health system factors associated with gonorrhea and chlamydia testing and case rates among women Veterans in the Veterans Health Administration (VHA) in 2019.

**Design:**

We performed a retrospective cohort study of all women Veterans in VHA care between January 1, 2018, and December 31, 2019.

**Participants:**

Women Veteran patients were identified as receiving VHA care if they had at least one inpatient admission or outpatient visit in 2019 or the preceding calendar year.

**Key Results:**

Among women under age 25, 21.3% were tested for gonorrhea or chlamydia in 2019. After adjusting for demographic and other health factors, correlates of testing in women under age 25 included Black race (aOR: 2.11, CI: 1.89, 2.36), rural residence (aOR: 0.84, CI: 0.74, 0.95), and cervical cancer screening (aOR: 5.05, CI: 4.59, 5.56). Women under age 25 had the highest infection rates, with an incidence of chlamydia and gonorrhea of 1,950 and 267 cases/100,000, respectively. Incidence of gonorrhea and chlamydia was higher for women with a history of military sexual trauma (MST) (chlamydia case rate: 265, gonorrhea case rate: 97/100,000) and those with mental health diagnoses (chlamydia case rate: 263, gonorrhea case rate: 72/100,000.)

**Conclusions:**

Gonorrhea and chlamydia testing remains underutilized among women in VHA care, and infection rates are high among younger women. Patient-centered, system-level interventions are urgently needed to address low testing rates.

**Supplementary Information:**

The online version contains supplementary material available at 10.1007/s11606-022-07578-2.

## INTRODUCTION

United States (US) rates of sexually transmitted infections (STI), especially gonorrhea and chlamydia, have increased dramatically over the past decade reaching record incidence in 2019.^1^ Women are vulnerable to both acute and chronic effects of STIs^2,3^ including pelvic inflammatory disease (PID), infertility, ectopic pregnancy, and pelvic pain.^4^ Gonorrhea and chlamydia infections also increase the risk of HIV acquisition.^3,4^

The Centers for Disease Control and Prevention (CDC) recommend annual screening for gonorrhea and chlamydia among sexually active women younger than 25 years and for women 25 years or older who are at increased risk of infection.^4^ This guideline is based on the high prevalence of these infections in young people and the risk of delayed diagnosis and treatment as a result of asymptomatic infections. Despite the availability of cost-effective, accurate, and noninvasive diagnostic tests,^5^ fewer than 50% of eligible women were screened for gonorrhea and chlamydia annually from 2001 to 2015^6^ in the US and fewer than 23% of 18–24-year-old women Veterans in VHA care were screened in 2019.^7^ No studies have described factors associated with gonorrhea or chlamydia testing among women in VHA care.

Women Veterans have many risk factors associated with STIs, including high rates of childhood sexual assault, military sexual trauma, and intimate partner violence.^8,9^ Trauma survivors are at risk for substance use disorders which can negatively impact safer sex practices, and may avoid accessing medical care that can be re-traumatizing.^10^ In addition, there are many known barriers to STI screening for both patients and clinicians.^11–14^ Strategies are needed to improve rates of guideline-concordant sexual health care among women Veterans within the Veterans Health Administration (VHA), especially gonorrhea and chlamydia screening.

The VHA health care network is the largest integrated civilian health care organization in the US, with 171 medical centers and 1,293 outpatient clinics across the country.^15^ In 2019, the VHA provided care to over 9 million enrollees, including 755,807 women.^16^ To improve screening and detection of STIs within the VHA health system, it is necessary to understand the current state of STI testing in women Veterans. Our study systematically examined patient characteristics and health care factors associated with gonorrhea and chlamydia testing and case rates among women Veterans in a US health care system.

## METHODS

### Study Design

We performed a retrospective cohort study of women Veterans in VHA care during 2019, defined as having at least one inpatient admission or outpatient visit in 2019 or the preceding calendar year. We extracted data from the VHA Corporate Data Warehouse (CDW), a data repository containing information from VHA electronic health records. The CDW data includes health care encounters, laboratory results, medications, diagnoses, and demographics.^17^ This analysis was conducted as part of a quality improvement effort and was considered exempt from institutional review board review. Drafting and submission of this manuscript complied with applicable VHA policies (VHA Program Guide 1200.21, *VHA Operations Activities That May Constitute Research*).

### Outcome Variable

To ascertain gonorrhea and chlamydia testing, we identified laboratory diagnostic tests performed via nucleic acid amplification or antigen detection during calendar year 2019. Gonorrhea and chlamydia testing is performed on a urine sample or a swab from the site of potential infection, and test sensitivity ranges from 86 to 100%.^18^ We included specimens from all the following sources in the analysis: vaginal, urine, oropharynx, and rectal specimens. Culture of gonorrhea or chlamydia is neither a recommended nor a widely used approach to screening or diagnosis.^19,20^

### Independent Variables

We included the following demographic variables: age, self-reported race, ethnicity, rurality, and homelessness status. We defined age as the age at the start of calendar year 2019. We defined rurality based on the patients’ most recent zip code. We identified patients as homeless if they experienced homelessness at any time between October 2015 and September 2019. We included additional variables at the patient level: HIV status, substance use disorder (SUD), mental health diagnosis, military sexual trauma (MST), intrauterine device contraception (in 2019), cervical cancer screening (performed during the 3-year period from 2017 to 2019), pregnant (in 2019), and HIV pre-exposure prophylaxis (PrEP) use (in 2019). We defined HIV based on the presence of viral markers and/or medication use related to HIV diagnosis prior to or through calendar year 2019. We included SUD diagnosis codes reported October 2018–September 2019, and any mental health diagnosis prior to or through calendar year 2019. We also included any MST documented in the electronic health record (EHR) as of the time of the study (see Supplemental Table: Variable Descriptions).

We examined patients’ health care contacts in 2019, defined as any encounter with the following specialties: gynecology, women’s health, primary care, clinical pharmacy, telehealth, and emergency department. To investigate regional variation in STI prevalence, we categorized patients’ most recently reported home address into census divisions. Census divisions are geographic units categorized into New England, Middle Atlantic, East North Central, West North Central, South Atlantic, East South Central, West South Central, Mountain, and Pacific (US Census Bureau, 2015).

### Statistical Analysis

We determined the proportion of women Veterans tested for chlamydia or gonorrhea at least once between January 1, 2019, and December 31, 2019, as well as the number of cases during this period. A new chlamydia or gonorrhea case was defined as any positive test result > 30 days after a previous positive result. Proportion of women tested and cases per 100,000 were determined for the overall cohort and within groups defined by patient health and demographic factors. Stratified results were also calculated for the subset of women Veterans under age 25. We fit a multivariable logistic regression model to examine the association between patient factors and testing for gonorrhea and chlamydia among women under age 25, the demographic group in our cohort that has an indication for annual screening. We conducted all analyses in SAS V9.4 using an alpha level of 0.05.

## RESULTS

### Testing

Overall, a cohort of 585,818 women Veterans of all ages were in VHA care in 2019, and 8.9% were tested in VHA for gonorrhea or chlamydia (Table 1). As compared with other racial groups, Black women had the highest rates of testing (13.0%). Women living in urban settings had higher rates of testing (10.1%) as compared with those living in rural areas (5.9%). Compared with all other types of care, women who saw a gynecologist had the highest rate of testing (29.7%). Women living in the South Atlantic census division had a higher rate of testing (10.4%) as compared with other census divisions.

**Table 1:** Gonorrhea and Chlamydia Testing and Rates of Infection for Women Veterans In-care in 2019

**Variable**	**Value**	**Women Veterans in-care** ***N***	**Tested for chlamydia or gonorrhea in 2019** ***n*** **(%)**	**Chlamydia cases per 100,000**	**Gonorrhea cases per 100,000**
All	All	585,818	52,315 (8.9)	222	57
Age group^1^	18–24	16,102	3,428 (21.3)	1,950	267
	25–29	43,720	8,666 (19.8)	956	194
	30–39	129,192	19,614 (15.2)	331	102
	40–49	115,227	11,219 (9.7)	84	43
	50+	281,544	9,378 (3.3)	16	8
Race	White	333,569	22,877 (6.9)	152	36
	Black	174,276	22,722 (13)	341	93
	Asian American	8,936	1,030 (11.5)	336	67
	Native American	6,376	596 (9.3)	314	78
	Hawaiian/Pacific Islander	6,429	620 (9.6)	311	62
	Unknown race	46,944	3,303 (7)	211	51
	Multiracial	9,288	1,167 (12.6)	366	97
Ethnicity	Hispanic ethnicity	45,742	5,975 (13.1)	400	92
	Non-Hispanic ethnicity	540,076	46,340 (8.6)	207	54
Rurality	Urban	425,763	42,846 (10.1)	261	68
	Rural/highly rural	153,935	9,133 (5.9)	118	24
	Unknown	6,120	336 (5.5)	163	49
HIV status	Living with HIV	1,143	385 (33.7)	700	350
	Not living with HIV	584,675	51,930 (8.9)	222	56
Living status	Experiencing homelessness	38,524	6,559 (17)	514	265
	Not experiencing homelessness	547,294	45,756 (8.4)	202	42
Substance use disorder	Yes	32,373	5,477 (16.9)	457	241
	No	553,445	46,838 (8.5)	209	46
Mental health diagnosis	Yes	413,840	43,677 (10.6)	263	72
	No	171,978	8,638 (5)	124	20
Military sexual trauma	Yes	15,482	1,782 (11.5)	265	97
	No	570,336	50,533 (8.9)	221	55
Health care contact	Gynecology	66,188	19,640 (29.7)	749	157
	No gynecology	519,630	32,675 (6.3)	155	44
	Women’s health	189,180	31,628 (16.7)	408	95
	No women’s health	396,638	20,687 (5.2)	134	38
	Primary care	400,230	39,291 (9.8)	252	67
	No primary care	185,588	13,024 (7)	158	34
	Clinical pharmacy	120,321	13,692 (11.4)	253	77
	No clinical pharmacy	465,497	38,623 (8.3)	215	51
	Telehealth	73,058	8,185 (11.2)	241	58
	No telehealth	512,760	44,130 (8.6)	220	56
	Emergency department	118,136	19,105 (16.2)	453	139
	No emergency department	467,682	33,210 (7.1)	164	36
IUD contraception	Using IUD contraception	30,541	11,048 (36.2)	1,418	229
	Not using IUD contraception	555,277	41,267 (7.4)	157	47
Cervical cancer screening	Screened for cervical cancer	195,567	39,508 (20.2)	494	116
	Not screened for cervical cancer	390,251	12,807 (3.3)	86	27
Pregnancy	Yes	20,121	3,355 (16.7)	631	149
	No	565,697	48,960 (8.7)	208	53
PrEP	Using PrEP	160	138 (86.3)	3,125	4,375
	Not using PrEP	585,658	52,177 (8.9)	222	55
Census division	New England	16,260	1,325 (8.1)	142	31
	Middle Atlantic	36,100	3,693 (10.2)	219	44
	East North Central	59,422	4,632 (7.8)	150	44
	West North Central	34,088	1,915 (5.6)	147	56
	South Atlantic	178,598	18,485 (10.4)	257	65
	East South Central	42,483	3,075 (7.2)	177	64
	West South Central	89,289	8,335 (9.3)	262	74
	Mountain	52,618	3,915 (7.4)	230	34
	Pacific	68,162	6,493 (9.5)	235	51
	Other	8,798	447 (5.1)	148	34

^1^*N*=33 with unknown age were excluded

Of women in VHA care in 2019, 16,102 women were 18–24 years old, of whom 3,428 (21.3%) were tested for gonorrhea or chlamydia at least once during the year (Table 2). Among women under 25 years of age, Black women had a higher rate of testing (30.6%) as compared with all other racial groups. Women under age 25 living in urban settings had a higher rate of testing (22.7%) as compared with those living in rural areas (16%). Among women under age 25, those who had a contact with gynecology during 2019 had the highest rate of testing (55.4%) as compared with other types of health care contacts. Women under age 25 residing in New England had the highest rate of testing (28.5%) as compared with all other census divisions.

**Table 2:** Gonorrhea and Chlamydia Testing and Rates of Infection in Women Veterans 18–24 Years of Age (2019)

**Variable**	**Value**	**Women Veterans (<25)** ***N***	**Tested for chlamydia/gonorrhea** ***n*** **(%)**	**Chlamydia cases per 100,000**	**Gonorrhea cases per 100,000**
All	All	16,102	3,428 (21.3)	1,950	267
Race	White	8,306	1,600 (19.3)	1,421	108
	Black	4,248	1,302 (30.6)	3,508	659
	Asian American	293	63 (21.5)	1,024	341
	Native American	183	27 (14.8)	1,639	0
	Hawaiian/Pacific Islander	234	42 (17.9)	1,282	427
	Unknown race	2,367	293 (12.4)	1,183	169
	Multiracial	471	101 (21.4)	2,123	0
Ethnicity	Hispanic ethnicity	2,613	601 (23)	2,181	77
	Non-Hispanic ethnicity	13,489	2,827 (21)	1,905	304
Rurality	Urban	12,689	2,879 (22.7)	2,104	315
	Rural/highly rural	3,192	511 (16)	1,347	63
	Unknown	221	38 (17.2)	1,810	453
Living status	Experiencing homelessness	610	208 (34.1)	3,771	1,312
	Not experiencing homelessness	15,492	3,220 (20.8)	1,878	226
Substance use disorder	Yes	650	238 (36.6)	2,923	462
	No	15,452	3,190 (20.6)	1,909	259
Mental health diagnosis	Yes	9,494	2,600 (27.4)	2,496	390
	No	6,608	828 (12.5)	1,165	91
Military sexual trauma	Yes	304	79 (26)	2,961	329
	No	15,798	3,349 (21.2)	1,931	266
Health care contact	Gynecology	2,406	1,332 (55.4)	4,447	707
	No gynecology	13,696	2,096 (15.3)	1,511	190
	Women’s health	4,599	1,918 (41.7)	3,979	587
	No women’s health	11,503	1,510 (13.1)	1,139	139
	Primary care	10,659	2,631 (24.7)	2,242	319
	No primary care	5,443	797 (14.6)	1,378	165
	Clinical pharmacy	1,953	720 (36.9)	3,840	512
	No clinical pharmacy	14,149	2,708 (19.1)	1,689	233
	Telehealth	1,468	432 (29.4)	2,112	273
	No telehealth	14,634	2,996 (20.5)	1,934	267
	Emergency department	2,987	1,246 (41.7)	4,587	703
	No emergency department	13,115	2,182 (16.6)	1,350	168
IUD contraception	Using IUD contraception	2,515	1,240 (49.3)	5,368	557
	Not using IUD contraception	13,587	2,188 (16.1)	1,317	213
Cervical cancer screening	Screened for cervical cancer	4,639	2,310 (49.8)	4,570	561
	Not screened for cervical cancer	11,463	1,118 (9.8)	890	148
Pregnancy	Yes	1,632	394 (24.1)	2,206	429
	No	14,470	3,034 (21)	1,921	249
PrEP	Using PrEP	4	3 (75)	--1	--1
	Not using PrEP	16,098	3,425 (21.3)	1,938	267
Census division	New England	390	111 (28.5)	2,308	0
	Middle Atlantic	972	226 (23.3)	1,852	617
	East North Central	1,478	292 (19.8)	1,827	203
	West North Central	795	141 (17.7)	1,006	252
	South Atlantic	4,816	1,100 (22.8)	2,243	291
	East South Central	1,013	202 (19.9)	1,974	296
	West South Central	2,565	540 (21.1)	1,988	351
	Mountain	1,441	259 (18)	1,804	139
	Pacific	2,332	509 (21.8)	1,758	129
	Other	300	48 (16)	2,000	333

^1^Rate per 100,000 not calculated due to small *N*

Several patient demographic and health factors were significantly associated with testing during 2019 among women under age 25 in a multivariable logistic regression model (Table 3). Black women had higher odds of being tested as compared with Whites (aOR: 2.11, CI: 1.89, 2.36). Hispanic women also had higher odds of being tested as compared with non-Hispanic women (aOR: 1.30, CI: 1.15, 1.49). Women living in rural areas had lower odds of being tested for gonorrhea or chlamydia relative to those in urban areas (aOR: 0.84, CI: 0.74, 0.95). Women under age 25 with substance use disorder (SUD) (aOR: 1.37, CI: 1.11, 1.68) or mental health diagnosis (aOR: 1.20, CI: 1.08, 1.34) had higher odds of being tested for gonorrhea or chlamydia compared with those not experiencing SUD or mental health diagnosis.

**Table 3:** Logistic Regression: Probability of Gonorrhea/Chlamydia Screening in 2019, Women Veterans 18–24 Years of Age, Independent Variables with *p*<0.05

**Variable**	**Value**	**Adjusted OR**	**95% CI**	***p*<0.05**
Race (Ref: White)				
	Black	2.11	1.89, 2.36	*
	Asian American	1.05	0.74, 1.49	
	Other	0.83	0.61, 1.13	
	Unknown race	0.95	0.81, 1.12	
	Multiracial	1.22	0.93, 1.60	
Ethnicity (Ref: Non-Hispanic)				
	Hispanic ethnicity	1.30	1.15, 1.49	*
Rurality (Ref: Urban)				
	Rural/highly rural	0.84	0.74, 0.95	*
	Unknown	4.18	1.69, 10.35	*
Substance use disorder (Ref: Not experiencing SUD)				
	SUD	1.37	1.11, 1.68	*
Mental Health Diagnosis (Ref: No mental health diagnosis)				
	Mental health diagnosis	1.20	1.08, 1.34	*
Health care contact				
Gynecology (Ref: Not seen by gynecologist)		2.86	2.54, 3.22	*
Women’s health (Ref: Not seen by women’s health)		2.87	2.59, 3.17	*
Primary care (Ref: Not seen by primary care)		1.89	1.70, 2.11	*
Clinical pharmacy (Ref: Not seen by pharmacy)		1.26	1.11, 1.44	*
Emergency department (ED) (Ref: Not seen by ED)		2.04	1.84, 2.28	*
IUD contraception (Ref: Not using IUD contraception)				
	Using IUD contraception	1.67	1.48, 1.88	*
Cervical cancer screening (Ref: No cervical cancer screening)				
	Received cervical cancer screening	5.05	4.59, 5.56	*
Pregnancy (Ref: Not pregnant)				
	Pregnant	0.73	0.63, 0.85	*
US census division (Ref: South Atlantic)				
	New England	1.70	1.28, 2.26	*
	Middle Atlantic	0.90	0.73, 1.10	
	East North Central	0.90	0.75, 1.08	
	West North Central	0.89	0.70, 1.13	
	East South Central	0.89	0.72, 1.09	
	West South Central	0.88	0.77, 1.02	
	Mountain	0.86	0.72, 1.04	
	Pacific	1.13	0.98, 1.32	
	Other	0.21	0.10, 0.48	*

*Indicates *p*<0.05

Region of residence and health care contact were significantly associated with testing after adjusting for covariates in the model. Women under age 25 who had contact with gynecology (aOR: 2.86, CI: 2.54, 3.22), women’s health primary care (aOR: 2.87, CI: 2.59, 3.17), primary care (aOR: 1.89, CI: 1.70, 2.11), clinical pharmacy (aOR: 1.26, CI: 1.11, 1.44), or the emergency department (aOR: 2.04, CI: 1.84, 2.28) had higher odds of being tested, with each group being relative to women not having the type of health care contact. Women under age 25 who had cervical cancer screening during 2017–2019 (aOR: 5.05, CI: 4.59, 5.56) had higher odds of being tested for gonorrhea or chlamydia as compared with women who did not have cervical cancer screening. Women under age 25 who were pregnant in 2019 had lower odds of being tested as compared with women who were not pregnant (aOR: 0.73, CI: 0.63, 0.85). In addition, women under age 25 using an IUD had higher odds of being tested as compared with those not using an IUD (aOR: 1.67, CI:1.48, 1.88). Women under age 25 living in New England had higher odds of being tested for gonorrhea or chlamydia as compared with women living in the South Atlantic.

### Case Rates

Overall, we identified 1,303 chlamydia infections and 331 gonorrhea infections (Table 1). Across all women in the cohort, the overall positivity rate was 2.2% for chlamydia and 0.6% for gonorrhea. The overall incidence of chlamydia and gonorrhea in 2019 was 222 and 57 cases per 100,000, respectively. Women Veterans aged 18–24 had the highest infection rates, with an incidence of chlamydia and gonorrhea of 1,950 and 267 cases per 100,000, respectively. For both chlamydia and gonorrhea, incidence was highest among multiracial women as compared with all other racial groups. Incidence of both gonorrhea and chlamydia was higher among women experiencing mental health conditions as compared with those not experiencing mental health conditions. Incidence of both gonorrhea and chlamydia was higher for women with a history of military sexual trauma as compared with women without history of military sexual trauma. Over a third of chlamydia cases (35.2%) and gonorrhea cases (35.0%) occurred in women who resided in the South Atlantic census division. Similarly, maps of gonorrhea and chlamydia case rates by state indicate that case rates are generally higher in Southern states.

## DISCUSSION

In our retrospective cohort study of factors associated with gonorrhea and chlamydia among women Veterans in-care in 2019, the rate of testing in women under age 25 was 21.3%. Higher incidence of both gonorrhea and chlamydia was observed in women under age 25, those living in urban settings, those with a history of military sexual trauma, and those with a mental health diagnosis. Among women under 25, Black women Veterans and those living in urban settings had significantly higher adjusted odds of being tested compared with White women and rural dwellers. Additionally, women who underwent cervical cancer screening or had contact with gynecology, women’s health, or primary care providers had significantly higher adjusted odds of being tested.

Higher incidence of gonorrhea and chlamydia among women Veterans with a history of military sexual trauma and those experiencing mental health diagnoses is consistent with known risk factors for this population. As a population, women Veterans experience a complex constellation of mental health diagnoses, sexual trauma, and gynecologic sequela known to be associated with STIs. Among women in the general population, a history of sexual assault is associated with higher lifetime risk for chlamydia.^21^ Women Veterans are more likely than non-Veterans to have experienced more severe and prevalent sexual violence across the lifespan, including childhood sexual assault, MST/assault, and sexual violence in their intimate relationships.^22^ As many as one-third of women Veterans experience MST,^23^ and over 18% self-report experiencing intimate partner violence in the last 12 months.^24^ Women Veterans are also more likely to experience mental health challenges such as post-traumatic stress disorder (PTSD) and depression, which can lead to substance use that may impede safe sex practices.^25,26^ This added burden of trauma and mental health diagnoses highlights the importance of early detection and treatment for gonorrhea and chlamydia.

Rates of gonorrhea and chlamydia testing among women ages 18–24 using VHA for any health care are lower in VHA than those reported in the general US population. The 2017–2019 CDC National Survey of Family Growth, which documents self-reported data from a nationally representative sample of US women, indicates that 26.2% of women under age 25 report having had chlamydia testing in the last year.^27^ According to the Health Effectiveness Data and Information Set, which relies on data from health insurance claims, gonorrhea and chlamydia screening occurred in 49.5% of women who met the definition of “sexually active” according to prescription data and administrative variables as of 2015.^6^ Other studies have documented that Black women are more likely to be tested for chlamydia than White women,^28,29^ and that clinics serving a higher proportion of Black women have higher rates of testing, which may be related to provider bias. Prior studies have documented lower rates of testing for chlamydia among women treated in rural settings.^12^ Moreover, as compared with clinicians who practice in urban settings, clinicians who practice in rural settings report more difficulties adhering to recommended preventative screening guidelines including assuring confidentiality and establishing a therapeutic alliance.^30^ Site and location of clinics may be associated with availability of reproductive care for female Veterans, and previous studies have noted that non-metropolitan VHA clinics were less likely to have gynecology on site.^31^

Several patient barriers have been reported to influence the use of STI screening. For example, lack of symptoms or poor awareness of the long-term implications of infection may hamper screening. Some women may avoid testing due to stigma or the time commitment associated with visiting a provider.^10,11^ Military sexual trauma among female Veterans may cause delayed access to reproductive health care.^32^

Clinician-related barriers to screening include lack of disease-related knowledge, lack of confidence, workload and time constraints, and lack of awareness of testing guidelines.^12,13^ Various interventions have successfully addressed these issues including provider education, skill-building, and clinical decision support through the EHR.^14^ As a large integrated health care system, the VHA is well-positioned to implement a national clinical decision support tool into the EHR to prompt and educate providers while they interact with patients. Effective CDS tools to improve GC/CT screening have incorporated an alert that displays in a specific area of the electronic medical record (EMR) as a notification that is visible whenever the chart of an eligible patient is accessed. Studies indicate that clinical decision support (CDS) tools can produce a 20–30% increase in rates of gonorrhea and chlamydia screening in primary care settings.^14,33^

Our findings indicate that women who had cervical cancer screening and had contact with a gynecologist, women’s health, or primary care physician were more likely to be tested as compared with those who did not have contact. Because cervical cancer screening is often done concomitantly with gonorrhea and chlamydia testing, there is an association between these screening practices. Studies indicate that when cervical cancer screening is done less frequently, screening for gonorrhea and chlamydia declines.^34,35^ Further, prior research indicates that obstetrician-gynecologists are more likely to regularly take a sexual history and offer STI counseling and screening.^3,36^ Since primary care providers are ideally positioned to screen and treat STIs, but tend to report more barriers to screening,^37^ it is critical to enhance support for primary care providers in delivering preventive, guideline-concordant care.

This study is a robust and comprehensive analysis of gonorrhea and chlamydia testing among female Veterans, but subject to several important limitations. Given that some testing in women under age 25 was ordered in response to symptoms or history, rather than purely for screening purposes, we could not ascertain screening rates in this group. Second, we examined data on patients’ health care contacts over the past year but do not report data regarding the specialty of the clinician who ordered the laboratory test. Third, our data lacked information on sexual activity and sexual risk behavior, which are factors known to be associated with gonorrhea and chlamydia screening and infection and included in the CDC recommendation for annual screening. Finally, we lack access to STI testing performed outside the VHA (e.g., through public health departments or STI clinics). Importantly, obstetrical care is typically outsourced by the VHA and results of prenatal STI testing are not available in VHA’s administrative data resources. It was, therefore, not surprising that our adjusted regression model of women under age 25 showed that pregnant women were less likely to be tested as compared with those who were not pregnant.

Assessment of gonorrhea and chlamydia testing among female Veterans indicates that testing remains underused and that infections are particularly high among younger female Veterans. Future studies are needed to delve deeper into the facility-level characteristics that may promote or hinder testing and into the sequelae of STIs in female Veterans. Further efforts are needed to develop patient-centered, system-level interventions to address low gonorrhea and chlamydia testing rates.

## Supplementary Information

ESM 1 (DOCX 17 kb)
