# Supplementary material for: Gonorrhea and Chlamydia Testing and Case Rates Among Women Veterans in the Veterans Health Administration
Source: J Gen Intern Med. 2022 Aug 30;37(Suppl 3):706–13. doi: 10.1007/s11606-022-07578-2 (PMC9481769; doi:10.1007/s11606-022-07578-2)
Supplement: Supplementary file 1 — (DOCX 17 kb) [file 11606_2022_7578_MOESM1_ESM.docx]

| **Supplemental Table: Variable Definitions** | |
| --- | --- |
| **Variable** | **Definition** |
| Age | Age at the start of CY 2019. |
| Rurality | Based on most recent home address of individuals. |
| HIV status | Individuals were defined as living with HIV if they met any of the following criteria in VHA administrative data prior to or through CY 2019:   1. Positive HIV antibody combined with positive confirmatory testing, 2. Positive HIV viral load, 3. Prescribed an HIV antiretroviral medication for ≥31 continuous days within the VHA, or 4. HIV included on their problem list. |
| Experiencing homelessness | Individuals were considered to be experiencing homelessness if touched by one of the VHA’s Homeless Services during October 2016 – September 2019.   1. Veterans currently living in a VA funded community-based residential homeless program. 2. Those Veterans who have screened positive to the Homeless Screening Clinical Reminder and indicated that their current living situation is on the streets or in a shelter. 3. Veterans who have had a Veterans Justice Program encounter. 4. Veterans who have had a Health Care for Homeless Veterans outreach encounter. |
| Substance use disorder | Individuals with an active diagnosis in CY 2019 based on ICD-10 codes for alcohol use disorder, cocaine use disorder, opioid use disorder, or other stimulant use disorder. |
| Mental health diagnosis | Individuals with a diagnosis prior to or through CY 2019 based on ICD-9 and ICD10 codes for anxiety disorder, bipolar disorder, depressive disorder, post-traumatic stress disorder, schizophrenia, or other mental health disorder. |
| Military sexual trauma | Self-reported MST documented in the individual’s electronic health record as of April 2021. |
| Health care contact | Outpatient visits during CY 2019 based on VHA visit codes for gynecology, women’s health, primary care, clinical pharmacy, telehealth, or emergency department. |
| IUD contraception | Based on ICD-10 or CPT codes during CY 2019. |
| Cervical cancer screening | Based on cytopathology specimens during 2017-2019. |
| Pregnancy | Based on ICD-10 codes during CY 2019. |
| PrEP | Individuals were considered to have received pre-exposure prophylaxis to prevent HIV infection if they had been prescribed an HIV antiretroviral medication for ≥31 continuous days within the VHA during CY 2019. |
| Census division | Based on most recent home address of individuals. Geographic units are categorized into New England, Middle Atlantic, East North Central, West North Central, South Atlantic, East South Central, West South Central, Mountain, and Pacific (US Census Bureau, 2015). |
